# Modulation of response times in early-stage Parkinson’s disease during emotional processing of embodied and non-embodied stimuli

**DOI:** 10.1038/s41598-024-63701-9

**Published:** 2024-06-06

**Authors:** Alessandro Botta, Elisa Pelosin, Giovanna Lagravinese, Roberta Marchese, Francesca Di Biasio, Gaia Bonassi, Sara Terranova, Elisa Ravizzotti, Martina Putzolu, Susanna Mezzarobba, Carola Cosentino, Alessio Avenanti, Laura Avanzino

**Affiliations:** 1 IRCCS Ospedale Policlinico San Martino, 16132 Genoa, Italy; 2 Department of Neuroscience, Rehabilitation, Ophthalmology, Genetics, Maternal and Child Health (DINOGMI), University of Genoa, 16132 Genoa, Italy; 3 Department of Experimental Medicine (DIMES), Section of Human Physiology, University of Genoa, Viale Benedetto XV 3, 16132 Genoa, Italy; 4 Centro Di Neuroscienze Cognitive and Dipartimento Di Psicologia, Campus Cesena, Alma Mater Studiorum-University of Bologna, Cesena, Italy; 5 Centro de Investigación en Neuropsicología y Neurociencias Cognitivas, Universidad Católica del Maule, Talca, Chile

**Keywords:** Parkinson’s disease, Embodiment, Emotion, Response time, Cognitive neuroscience, Emotion, Parkinson's disease

## Abstract

Valence (positive and negative) and content (embodied vs non-embodied) characteristics of visual stimuli have been shown to influence motor readiness, as tested with response time paradigms. Both embodiment and emotional processing are affected in Parkinson’s disease (PD) due to basal ganglia dysfunction. Here we aimed to investigate, using a two-choice response time paradigm, motor readiness when processing embodied (emotional body language [EBL] and emotional facial expressions [FACS]) vs non-embodied (emotional scenes [IAPS]) stimuli with neutral, happy, and fearful content. We enrolled twenty-five patients with early-stage PD and twenty-five age matched healthy participants. Motor response during emotional processing was assessed by measuring response times (RTs) in a home-based, forced two-choice discrimination task where participants were asked to discriminate the emotional stimulus from the neutral one. Rating of valence and arousal was also performed. A clinical and neuropsychological evaluation was performed on PD patients. Results showed that RTs for PD patients were longer for all conditions compared to HC and that RTs were generally longer in both groups for EBL compared to FACS and IAPS, with the sole exception retrieved for PD, where in discriminating fearful stimuli, RTs for EBL were longer compared to FACS but not to IAPS. Furthermore, in PD only, when discriminating fearful respect to neutral stimuli, RTs were shorter when discriminating FACS compared to IAPS. This study shows that PD patients were faster in discriminating fearful embodied stimuli, allowing us to speculate on mechanisms involving an alternative, compensatory, emotional motor pathway for PD patients undergoing fear processing.

## Introduction

Parkinson’s disease (PD) is a neurodegenerative disease characterized by death of dopaminergic neurons in the substantia nigra. Besides the well-known motor symptoms, patients with PD frequently experience non-motor symptoms, including cognitive and affective disturbances. Particularly, anxiety and emotion dysregulation are prevalent in PD, with up to 40% of PD patients suffering from depression^1^.

A novel line of research is focused on the influence of non-motor aspects, such as emotions, on motor behaviour in PD^2^. Impairments of facial expressions, upper limb performance and gait has been observed in PD in relation to emotional processing^3–7^. Indeed, it has been demonstrated that both spontaneous and posed facial expressions are altered and diminished in PD patients^6,8^. Evidence showed that emotional disturbances arising from affective disorders (such as anxiety and depression) could contribute to gait slowness, freezing of gait and a general worsening of motor performance in people with PD^3,5,7,9^. Specifically, the observation of stress-evoking, aversive emotional stimuli has been shown to significantly reduce fine motor control abilities (in terms of increased variability of force output while performing an isometric precision grip) in PD patients^4^.

Emotional processing can be studied with different set of stimuli, ranging from non-embodied (i.e., stimuli depicting emotional scenes taken from the International Affective Picture System [IAPS] database) to embodied stimuli (i.e., facial expression or images depicting emotional body postures). Recently we have shown that in healthy subjects the readiness of motor system is influenced by the content of the images. Indeed we found a behavioural advantage, represented by a decrease in response time in recognizing fearful emotional body language (EBL) with respect to IAPS images, probably thanks to the automatic activation of central nervous system structures orchestrating a defensive reaction^10^. These findings have been explained considering the ‘embodied simulation theory’ postulating that the exposure to embodied visual stimuli would automatically trigger action simulation in the mind of the observer, thereby originating a ‘feeling of movement’ modulated by the mirror neuron system and influencing the readiness of the motor system^10^. Indeed, EBL has the advantage of conveying not only emotional, but also motor information from limbs and axial muscles, hence implicating mechanisms of motor mimicry and embodiment^11^. The same is for facial expressions, conveying emotional and motor information from facial muscles.

Evidence on embodiment and imitative mechanisms of PD patients are nowadays inconclusive, showing contrasting results indicating on one hand preserved motor mimicry mechanisms (as an example see^12^) and on the other deficits in engaging the motor system during action observation^13^, in recognizing emotional facial expression, especially in presence of hypomimia^14,15^ and in words production^16^. Moreover, related to embodiment, the processing of emotional embodied stimuli shows contradictory evidence in PD patients, especially in terms of valence evaluation and accuracy, with some studies showing worse performance in PD patients, and others showing no differences between groups^17^.

Given the influence that emotional processing exerts on motor behaviour in PD^3–7^, we hypothesize that motor readiness will be influenced by the emotional content of the images. Furthermore, by following the ‘embodied simulation theory’, we postulate that altered motor function in PD (that is characterized by bradykinesia and rigidity) might interfere with the priming effects of embodied stimuli on motor readiness.

In this study we explored whether the response time in discriminating fearful and happy stimuli differed between embodied vs non-embodied set of stimuli in patients with early-stage PD. To this aim, participants were asked to discriminate the emotional stimulus with respect to the neutral one and we collected response times, as a proxy of the readiness of the motor system. The following three set of images were used: emotional body language (EBL), emotional facial expressions (FACS) and emotional scenes (IAPS). Following previous studies of our group^7,10,18,19^, we decided to include only fearful and happy visual stimuli. Indeed, we already showed that these emotions can modify different neurophysiological and behavioural aspects of healthy and pathological subjects, but to the best of our knowledge, there are no studies that directly compare these emotions in the three sets of stimuli used in this study in PD patients.

## Materials and methods

### Study design

We designed an observational, within-subjects, crossover, controlled study, where all participants had to complete three randomly selected sessions of a home-based two-alternative forced choice discrimination task.

### Participants

The experiment included a total of 25 patients diagnosed with Parkinson's disease (PD), of which 11 were females, with a mean age of 65.31 years (± 8.44) and an average Hoehn and Yahr (H&Y) score of 1.79 (± 0.47). Additionally, 25 healthy controls (HCs) of comparable age (mean age: 63.78 ± 5.61) were recruited, with 10 of them being females. All participants self-reported as right-handed. The PD patients were recruited from the Centre for Parkinson’s Disease at IRCCS San Martino of Genoa (Italy). Each participant provided informed consent digitally. The experimental procedure received approval from the ethics committee at the University of Genoa (protocol number 2020/16) and was conducted in accordance with the principles outlined in the Declaration of Helsinki concerning experiments involving human participants.

### Eligibility criteria

PD patients had to fulfil the following inclusion criteria: (i) diagnosis of idiopathic PD (according to the United Kingdom Parkinson’s Disease Society Brain Bank criteria), (ii) Hoehn and Yahr stage 1–2 and (iii) Mini Mental State Examination (MMSE) score > 24. Patients were excluded in presence of (i) history of other neurological disorders (except PD) and (ii) visual or orthopaedic impairments that could interfere with a correct task performance. HC were enrolled if they met the following inclusion criteria: (i) Mini Mental State Examination (MMSE) score > 24; (ii) clinical history not suggestive for visual or orthopaedic impairments that could hinder task performance.

### Clinical assessment

PD severity was rated via the MDS-unified parkinson disease rating scale, part III (MDS-UPDRS III), while cognitive functions were evaluated with the PD-cognitive rating scale (PD-CRS). An affective evaluation was also performed via specific scales such as the hamilton depression rating scale (HAM-D) and the hamilton anxiety rating scale (HAM-A). All patients were currently undergoing dopaminergic therapy and were instructed to carry out the experiment within a time frame of one to two hours following their anti-parkinsonian medication administration.

### Visual stimuli

Emotional pictures were taken from three different databases: EBL from the experiments of Borgomaneri and colleagues^10,18,20^, emotional scenes from the IAPS database^21^ and facial expressions from the Ekman’s Facial Action Coding System (FACS) test^22^.

A total of 90 slides were used in the experimental sessions, divided as follow: 30 for EBL, 30 for IAPS and 30 for FACS. Each slide included two images, one emotional (fearful or happy) and one non-emotional, and of the 30 slides used in the experiments 15 depicted fear and 15 happiness. Emotional pictures allocation (i.e., right or left of the slide) was random, as the order of slides presentation.

### Tasks and experimental procedure

The experiment involved three sessions that participants carried out from their homes, one for each set of images. In each session, participants were required to download the task from the E-Prime Go online platform (Psychology Software Tools, Pittsburgh, PA, United States). They were instructed to perform the task on their personal computers in a quiet environment. The experiment was designed using E-Prime 3.0 software (Psychology Software Tools, Pittsburgh, PA, United States). During each session, the slides were displayed on the screen for 2000 ms, with a fixation cross appearing for 1500 ms between each visual stimulus. The order of the three sessions was randomized. Participants were instructed to complete a two-alternative forced choice discrimination task, aiming to press either the left arrow key or the right arrow key as quickly as possible corresponding to the side of the slide that displayed the emotional image (i.e., fearful or happy). This task was used to calculate response times (RTs), which were measured in milliseconds as the time between the onset of the visual stimulus and the key-press. All participants received an online questionnaire to evaluate the valence and arousal associated to the visual stimuli after the three experimental sessions concluded. Valence and arousal were rated on a Likert scale spanning from 1 to 9, where a rating of 1 indicated ‘completely unpleasant,’ and 9 denoted ‘utterly pleasant’ for valence. Similarly, a rating of 1 signified ‘lack of arousal,’ while 9 indicated ‘high levels of arousal’ on the scale used for assessing arousal.

### Data analysis

#### Response time data

The RT data analysis was conducted using a repeated measures analysis of variance (RM ANOVA), with GROUP (PD and HC) as the between-subject factor and PICTURE (EBL, IAPS, and FACS) as the within-subject factor. This analysis was carried out separately for the 'Fear' and 'Happiness' visual stimuli. Any RTs that fell outside of the range of two standard deviations from their respective means were identified as outliers.

#### Correlation analysis

We also explored whether reaction times (RTs) in the discrimination task were influenced by several factors, including dopaminergic therapy (measured as levodopa equivalent daily—LEDD), disease duration in years, disease severity (assessed through H&Y stage and MDS-UPDRS III), levels of cortical and subcortical dysfunction (via PD-CRS subcortical and cortical sub-scores), anxiety (measured using HAM-A), and depression (evaluated with HAM-D). In the case of MDS-UPDRS III, we also conducted a correlation analysis to examine the relationship between RTs and its sub-items 3.2 (Facial expression) and 3.13 (Posture), taking into consideration the content of the embodied stimuli (FACS and EBL).

The non-parametric correlation analysis was carried out using the Spearman’s rank correlation method.

#### Valence and arousal

The data regarding valence and arousal were subjected to a RM ANOVA, conducted separately for each emotion. In this analysis, GROUP served as the between-subjects factor, while PICTURE was the within-subjects main effect. Subsequently, post hoc analysis was carried out, and significance levels were adjusted using the Bonferroni correction.

#### Accuracy

Accuracy was calculated as the percentage of the errors performed by both groups in the discrimination task, for each emotions (i.e., fear and happiness), A comparison between groups was then performed via a two tailed paired t-test, comparing the number of errors made in discriminating fearful and happy visual stimuli for each set of pictures.

## Results

### Participants

Twenty-two patients with PD and twenty-two HC entered in the analysis. Six participants (3 HC, 3 PD) were excluded, since RT data were outliers (mean ± 2 × SD) in one of the experimental conditions. The two groups were matched for age (p = 0.45) and gender (p = 0.65). Participants’ characteristics are reported in Table 1. To strengthen the reliability of our results, we performed a post-hoc power analysis on G*Power 3.1.9 with the following parameters: sample size 22; effect size = 0.924; α = 0.05. The result of the analysis showed a 1-β = 0.985, indicating an appropriate sample size for the study.

**Table 1:** Demographic, clinical and neuropsychological characteristics of patients with Parkinson’s disease and healthy subjects. In the last column of the table (P-value) it is possible to observe the results of the statistical analysis between groups performed for age and sex.

Demographic, clinical and neuropsychological characteristics.			
	PD	HC	P-value
Gender	(11M, 11F)	(12M, 10F)	p = 0.64

	Mean ± SD	Mean ± SD	
Age (years)	65.31 ± 8.44	63.78 ± 5.61	p = 0.45
Disease duration (years)	8.29 ± 4.07	-	-
Hoehn & Yahr (stage)	1.79 ± 0.47	-	-
LEDD (mg)	541.19 ± 228.10	-	-
UPDRS part III (score)	23.19 ±11.78	-	-
PD-CRS TOT (score)	99.10 ± 25.52	-	-
PD-CRS cortical (score)	31.39 ± 11.20	-	-
PD-CRS cortical (score)	72.43 ± 17.83	-	-
HAM-A	10.00 ± 6.86	-	-
HAM-D	12.95 ± 8.27	-	-

*PD* Parkinson’s disease, *HC* healthy control, *UPDRS* motor section of the movement disorder society unified Parkinson’s disease rating scale, *LEDD* levodopa equivalent daily dose, *PD-CRS* Parkinson’s disease cognitive rating scale, *HAM-A* hamilton anxiety rating scale, *HAM-D* hamilton depression rating scale.

### Response time data

**Figure 1:**
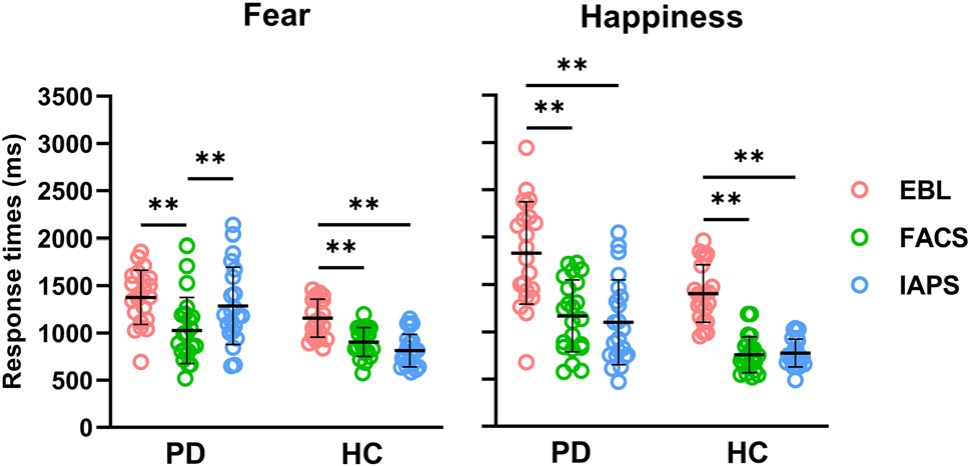
Response times *(RTs)*. The picture shows the mean RTs for both groups (*Parkinson’s disease, PD,* PD and *Healthy controls, HC) for* ‘Fear’ (left panel) and ‘Happiness’ (right panel). RT data for each subject are plotted with the thick black line indicating the mean value for the group and the respective standard deviation. Asterisks indicate significant post-hoc of the PICTURE*GROUP interaction (** = p < 0.01).

Results for RTs in the discrimination task are reported in Fig. 1.

For the fearful stimuli, the RM ANOVA revealed significant effects of GROUP (F_1, 42_ = 15.252; p < 0.001), PICTURE (F_2, 84_ = 35.724; p < 0.001) and a significant PICTURE × GROUP interaction (F_2, 84_ = 5.749; p = 0.048). Post hoc analysis of the GROUP main factor indicated that PD patients were generally slower than the control group in the discrimination task (p < 0.001). When examining the PICTURE main factor, it was observed that RTs were longer when the discrimination task involved EBL stimuli compared to FACS (p < 0.001) and IAPS (p < 0.001), with no significant difference between FACS and IAPS (p > 0.05). However, the post hoc analysis for the interaction effect revealed that RTs were longer for EBL compared to both FACS and IAPS in the control group (EBL vs FACS, p < 0.001; EBL vs IAPS, p < 0.001), but this difference was not observed in the PD patients' group. In PD patients, significantly shorter RTs were observed for FACS when compared to EBL (p < 0.001) and IAPS (p < 0.001), but no significant differences were found between EBL and IAPS (p > 0.05). Furthermore, in PD only RTs were shorter in the discrimination task involving FACs with respect to IAPS (p < 0.001), whereas in HC there was no difference between FACs and IAPS (p > 0.05).

For happy stimuli, RM ANOVA showed a significant effect of GROUP (F_1, 42_ = 20.789; p < 0.001) and PICTURE (F_2, 84_ = 84.681; p < 0.001), with no significant PICTURE × GROUP interaction (F_2, 84_ = 0.250; p = 0.779). Post hoc analysis of the GROUP main factor indicated that PD patients exhibited longer reaction times (RTs) than the control group in the RT discrimination task (p < 0.001).Furthermore, post hoc analysis of the PICTURE main factor indicated that RTs were longer when the discrimination task included EBL stimuli as compared to FACS (p < 0.001) and IAPS (p < 0.001) in both groups.

### Correlation analysis

**Figure 2:**
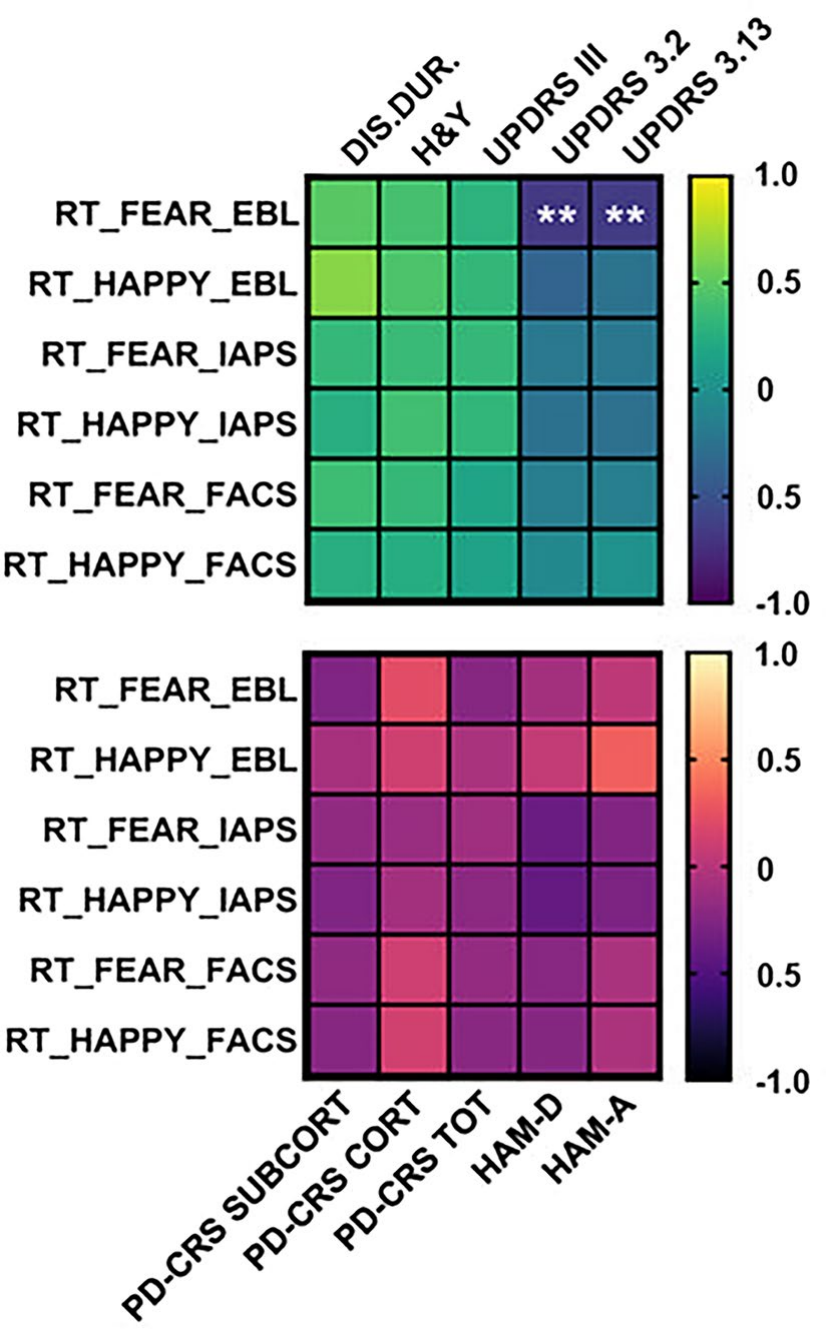
Correlation matrices. The pictures show the correlation matrices of RTs in all conditions and the clinical (above panel) and neuropsychological (below panel) characteristics of PD patients, corrected for multiple comparisons. As observable, the only two correlations lasting after correction are the ones between fearful RTs to EBL stimuli and the two subitems (i.e., 3.2 and 3.13) of the MDS-UPDRS III indicating the scores for ‘facial expression’ and ‘posture’ (far right in the above panel). Legend: *RT* response times, *FEAR* fearful emotional stimuli (‘Fear’ condition), *HAPPY* happy emotional stimuli (‘Happiness’ condition), *EBL* emotional body language, *IAPS* international affective picture system, *FACS* facial action coding system, *PD-CRS SUBCORT* subcortical subitems of the PD-CRS, *PD-CRS CORT* cortical subitems of PD-CRS, *PD-CRS TOT* summation of cortical and subcortical subitems of PD-CRS, *HAM-D* hamilton depression scale, *HAM-A* hamilton anxiety scale. ** = p < 0.01.

Correlation matrices between RTs and clinical and neuropsychological variables are shown Fig. 2.

Correlation analyses results were corrected for multiple comparisons following the method by Curtin and Schulz^23^. The significance value corrected for multiple comparisons was then p = 0.005. Results revealed a significant negative correlation between RTs, when discriminating fearful EBL, and the MDS-UPDRS III “facial expression” (question 3.2: r = −0.067, p = 0.002) and “posture” (question 3.13: r = −0.652, p = 0.003) subitems. RTs, when discriminating EBL, did not correlate with any of the other variables considered (clinical and neuropsychological). No significant correlations were found for RTs when discriminating IAPS and FACS and clinical and neuropsychological characteristics of PD patients.

### Valence and arousal scores

As for the valence and arousal scores, results are shown in Table 2. A total of twenty PD patients and 16 healthy controls consistently fulfilled the online questionnaire. Statistical analysis for valence in the ‘Fear’ condition showed a significant effect for PICTURE (F_2, 68_ = 16.695; p < 0.001) but not for GROUP and for the interaction effect (F < 1; p > 0.05). Specifically, valence scores were lower for IAPS stimuli in comparison to EBL (p < 0.001) and FACS (p < 0.001), while no significant differences were observable between EBL and FACS. Analysis on arousal for ‘Fear’ showed a significant effect for GROUP (F_2, 36_ = 8.779; p = 0.006) and PICTURE (F_2, 68_ = 32.252; p < 0.001), with no significant interaction effect. Post hoc analysis regarding GROUP showed that PD patients reported lower arousal compared to healthy controls. Post hoc analysis related to PICTURE demonstrated higher arousal for IAPS stimuli when compared to EBL (p = 0.001) and FACS (p = 0.006). As for the ‘Happiness’ condition, the statistical analysis on valence showed significant results for PICTURE (F_2, 68_ = 22.225; p < 0.001) but no significant effect for GROUP and for the interaction (F < 1; p > 0.05). Post hoc analysis revealed higher valence for IAPS stimuli compared to EBL (p < 0.001) and FACS (p < 0.001). In the data analysis for arousal there was a significant effect for GROUP (F_2, 68_ = 8.240; p = 0.007) and PICTURE (F_2, 68_ = 11.514; p < 0.001) with no significant interaction effect. In the ‘Happiness’ condition, PD patients reported lower arousal compared to healthy controls. Post hoc analysis related to PICTURE showed higher arousal for IAPS pictures compared to EBL (p < 0.001) and FACS (p < 0.001).

**Table 2:** Valence, arousal and accuracy. The table shows all values for valence and arousal retrieved by the stimuli evaluation from the questionnaires and the percentage of accuracy during the discrimination task. All values are reported as mean ± standard deviation. Accuracy values are reported as percentage of correct answers in the discrimination task.

Valence, arousal and accuracy							
		Fear			Happiness		
		EBL	IAPS	FACS	EBL	IAPS	FACS
PD	Valence	3.03 ± 1.24	1.72 ± 0.65	2.86 ± 1.03	6.45 ± 1.28	8.03 ± 0.75	6.94 ± 1.23
	Arousal	4.14 ± 2.31	6.04 ± 2.26	4.42 ± 2.22	4.42 ± 2.26	6.99 ± 1.61	5.59 ± 2.15
	Accuracy	95.31 ± 7.87	98.62 ± 6.16	99.08 ± 2.89	88.10 ± 12.52	98.62 ± 4.06	96.67 ± 5.19
HC	Valence	2.37 ± 0.60	1.89 ± 0.52	2.63 ± 0.94	6.92 ± 1.11	7.86 ± 0.71	7.21 ± 1.02
	Arousal	5.35 ± 1.56	7.18 ± 1.24	6.15 ± 1.69	6.30 ± 1.52	7.39 ± 0.82	6.60 ± 1.02
	Accuracy	95.00 ± 7.26	98.89 ± 4.16	97.50 ± 4.64	91.11 ± 12.42	98.88 ± 4.52	97.78 ± 5.33

### Accuracy

Accuracy data are reported in Table 2. We found an overall accuracy in the discrimination task of 96% for both PD patients and HCs (details can be found in Table 2). Statistical analysis showed no significant differences between groups in accuracy (all p > 0.05), with the only exception being for happy facial expressions, where a higher accuracy was retrieved for HC compared to PD (p < 0.01).

## Discussion

The aim of this study was to explore whether the response time, as a proxy of the readiness of the motor system, in discriminating emotional stimuli differed among different types of stimuli, characterized by the presence or not of embodiment in patients with early-stage PD. To this aim we investigated motor readiness when processing embodied (emotional body language [EBL] and emotional facial expressions [FACS]) vs non-embodied (emotional scenes [IAPS]) stimuli with neutral, happy, and fearful content.

The key findings are as follows: (i) Overall, individuals with PD displayed longer response times than healthy controls (HC) when discriminating both fearful (‘Fear’) and happy (‘Happiness’) stimuli.; (ii) In both PD and HC groups, when distinguishing ‘Happiness’ from non-emotional (‘Neutral’) stimuli, response times were prolonged when discriminating emotional body language (EBL) in comparison to facial action coding system (FACS) and international affective picture system (IAPS) stimuli; (iii) In HC, when distinguishing ‘Fear’ in contrast to ‘Neutral’ stimuli, response times were longer for EBL when compared to FACS and IAPS stimuli. However, in individuals with PD, while discriminating ‘Fear’ versus ‘Neutral’ stimuli, response times were longer when discriminating EBL only in comparison to FACS but not when compared to IAPS; (iv) Specifically in individuals with PD, when distinguishing ‘Fear’ stimuli versus ‘Neutral’ stimuli, response times were shorter for FACS compared to IAPS;(v) In individuals with PD, response times for discriminating fearful EBL stimuli exhibited a significant correlation with sub-items 3.2 and 3.13 of the MDS-UPDRS III, which pertain to ‘facial expression’ and ‘posture,’ respectively.

First, longer RTs in PD in all sets of pictures (‘Fear’ and ‘Happiness’), compared to HC may be ascribed to one of the main symptoms in PD, which is bradykinesia.

In terms of the emotional aspects of the stimuli, both the PD and HC groups exhibited comparable behaviour when exposed to stimuli associated with ‘Happiness.’ In both cases, they displayed longer RTs when distinguishing between EBL in comparison to IAPS and the FACS stimuli, with no significant difference observed between the latter two. This outcome aligns with our prior research, which examined RTs in distinguishing EBL in relation to IAPS^10^ and can be attributed to the characteristics of the stimuli. One potential interpretation for this result could be that the information conveyed by the arms and hands in EBL, which play a crucial role in accurately processing certain specific emotions^24,25^, are ambiguous in happy images. A closed fist, that is often present in ‘Happiness’ EBL stimuli, might be an indication of anger^24,26^, and hence, it might results in longer times for correctly interpreting the observed body posture. Given that the hands demand more attentional resources when interpreting the emotions conveyed through body language, and considering that our happy stimuli predominantly featured actors with closed fists in cheerful postures like jubilation or exultation, it is reasonable to speculate that the prolonged response times observed in the ‘Happy’ condition could be attributed to this incongruence between the body posture and hand gestures.

Differences between individuals with PD and HC emerged during the discrimination task involving ‘Fear’ stimuli. Our findings indicate that individuals with PD exhibit accelerated motor responses when faced with potential threats, as represented by fearful stimuli in the embodied set. This heightened response speed in PD led to non-significant differences in RTs when distinguishing ‘Fear’ EBL stimuli from ‘Fear’ IAPS stimuli, and even faster responses when discriminating between ‘Fear’ FACS stimuli from ‘Fear’ IAPS stimuli, in contrast to the responses of HC. Alternatively, we could interpret our findings as indicating that individuals with PD may struggle to recognize ‘Fear’ IAPS stimuli, as evidenced by their longer reaction times in response to these stimuli compared to ‘Fear’ FACS and similar reaction times to ‘Fear’ EBL stimuli (differently from what observed for happiness). However, this explanation is less likely, given that the analysis of valence scores did not reveal any significant PICTURE × GROUP interaction, indicating a similar ability for individuals with PD and HC in recognizing emotions across the three sets of stimuli. It’s important to highlight that, similar to our earlier study^10^, we specifically chose IAPS stimuli for the ‘Fear’ condition from a limited pool of images known to predominantly elicit fear, such as those depicting human attacks and accidents^27^. Secondly, in order to eliminate the majority of body movement cues, we made the deliberate choice to exclude any IAPS images showing entire human bodies engaged in actions. This decision ensured that we had a suitable set of images for direct comparison with the 'embodied' stimulus sets, namely FACS and EBL.

From our findings we can infer that embodied stimuli representing fear (i) are more rapidly processed in PD *OR* (ii) may induce a faster motor response in the discrimination task.

Related to the first hypothesis, there are data in the literature on early emotion processing in PD collected by means of event-related potentials (ERPs)^28^. Results showed that pictures of high compared to low emotional arousal were associated with a pronounced relative negative shift in the ERP waveform over parietal and occipital sites developing about 220 ms after picture onset. This early posterior negativity (EPN) did not differ between PD and control group, suggesting early emotion processing in PD is comparable to controls. In a successive work the authors investigated EPN in response to emotional compared to neutral facial expressions (angry, fearful, disgusted, sad, and happy)^29^. Results showed that in control subjects, the EPN of the ERP, which is thought to reflect early perceptual emotion discrimination, was larger in response to emotional compared to neutral facial expressions. In contrast, this emotional modulation of the EPN was absent in PD patients indicating impaired early emotion discrimination. Noteworthy, any difference was found related to latency of EPN, reflecting a processing time comparable between PD and controls. Taking into consideration these data, we consider unlikely that our results may be the consequence of faster processing of emotional stimuli. Indeed, our findings are specific to ‘Fear’ embodied stimuli, whereas ERP data indicated impaired early emotion discrimination for all emotional face expression. Furthermore, we found here a gain of performance in discriminating ‘Fear’ embodied stimuli and not the opposite.

Regarding the second hypothesis, i.e., that embodied stimuli representing fear induce a faster motor response in the discrimination task, we can discuss this hypothesis considering the effect that emotional stimuli exert on motor function in PD. In PD, it has been described since many years the ‘kinesia paradoxa’ phenomenon, defined as “the sudden transient ability of a patient with PD to perform a task he or she was previously unable to perform”^30^. Various theories have been developed to explain this phenomenon ranging from noradrenergic activation to activation of alternative motor pathways with respect to those normally activated during voluntary movement under non-emotional circumstances^31^. Particularly, activation of the limbic circuit of the basal ganglia, one of the multiple and segregated cortico-subcortical-cortical pathways involving the basal ganglia^32^, has been proposed^2^.

Hence, we can speculate that the ‘Fear’ embodied stimuli may induce a switch to the emotional basal ganglion module, making motor response (here, response times) faster.

This alternative ‘emotional’ motor pathway can play a compensatory role in PD to counteract dysfunctions induced by dopaminergic deficit in other basal ganglia motor pathways, making the motor aspects in PD so sensible to emotional distress^2^. The effectiveness of these affective compensatory mechanisms might be suggested by the negative correlations we found between RTs in discriminating ‘Fear’ EBL stimuli and the two MDS-UPDRS III subitems investigating facial mimicry and posture, apparently making the EBL set of stimuli particularly sensible to test motor readiness in response to emotional stimuli, possibly because of the amount of motor information conveyed by this set of stimuli.

Based on these results, we can hypothesize that PD patients with more severe axial symptoms are apparently more able to call into action the compensatory ‘emotional’ motor pathway compared to patients with less symptoms, especially when it comes to threatening stimuli processing. Following this line of reasoning we can also speculate that lost in this compensation may be responsible of worsening of motor symptoms as in the case of freezing of gait (FOG), that is largely sensible of emotional distress with worsening of FOG symptoms in relation to anxiety^3^.

Here, we recruited patients in their early clinical stage (phases 1 and 2 of Hoehn and Yahr scale) and no one experienced FOG. Following a hypothesis implying the loss of compensation of emotional alternative pathway in PD with FOG, RTs when discriminating ‘Fear’ embodied stimuli are not supposed to be speeded up in FOG patients, due to the loss of compensatory activity in the emotional alternative pathway. It would be interesting to address this hypothesis in future studies.

Some limitations of the present study should be considered. First, the experiment was conducted on-line and no direct monitoring from the experimenter was available. However, results were consistent, and outliers were excluded from the analysis. Second, this study included only patients in the early stage of the disease and to include patients in a more advanced stage (also with FOG) is required to confirm our hypothesis on the relation between RTs modulation with ‘Fear’ embodied stimuli and symptoms worsening. Third, in this study, consistently with other works of our group^7,10,18,19^, we choose only fear and happiness as emotions depicted in the visual stimuli. Future studies should investigate how other negative emotions such as anger or sadness can modify motor behavior, especially in presence of a neurodegenerative disease such as PD. Finally, recent findings showed that PD patients show a deficit in recognising disgusted facial expressions^29^, especially if in presence of hypomimia^14,15^. Disgust is considered one of the six basic emotions^33^ and is in the ‘group’ of the negative emotions; in this study we did find a significant correlation between hypomimia (item 3.2 of MDS-UPDRS III) and RTs, but no differences in terms of accuracy in the comparison with HC, hence indicating that hypomimia did not interfere with the emotional discrimination task (at least when it comes to fear and happiness). In any case, considering the aforementioned works, it would be interesting to address whether motor responses in PD are modulated by disgust processing.

In summary, we have demonstrated that in the context of a discrimination task involving ‘Fear’ embodied stimuli (FACS and EBL), motor responses were notably quicker in PD patients. This observation is substantiated by the shorter reaction times (RTs) for FACS compared to IAPS and the absence of a significant RT difference, despite the variance in complexity, for EBL in comparison to IAPS. We have put forth hypotheses regarding potential mechanisms that could underlie the engagement of an alternative emotional motor pathway, contributing to improved motor performance during ‘Fear’ processing.

## Data Availability

The data that support the findings of this study are available from the corresponding author upon reasonable request.
